# Engineering CHO cell metabolism for growth in galactose

**DOI:** 10.1186/1753-6561-5-S8-P119

**Published:** 2011-11-22

**Authors:** Natalia E Jiménez, Camila A Wilkens, Ziomara P Gerdtzen

**Affiliations:** 1 Centre for Biochemical Engineering and Biotechnology, Department of Chemical Engineering and Biotechnology, University of Chile, Santiago, 8370448, Chile; 2 Millennium Institute for Cell Dynamics and Biotechnology: a Centre for Systems Biology, University of Chile, Santiago, 8370448, Chile

## Background

Chinese hamster ovary (CHO) cells are one of the main hosts for industrial production of therapeutic proteins, owing to well-characterized technologies for gene transfection, amplification, and selection of high-producer clones. This has motivated the search for different strategies for the improvement of their specific productivity being one of the key points for this approaches the reduction of metabolic end-products like lactate and ammonia.

The use of different carbon sources has been an alternative solution for this problem, as they are metabolized more slowly than glucose leading to lower production of metabolic end-products [1]. Particularly, it has been observed that cultures in presence of glucose and galactose undergo a metabolic shift in which they are capable of remetabolize lactate. However, the specific growth rate is diminished due to a slower metabolism associated to the incorporation of galactose [2]. In addition, cells are unable to survive with galactose as their unique carbon source [3].

In this work we aim at identifying culture conditions that extend the culture's viability for tPA producing CHO cells in media with combined glucose and galactose as carbon sources. Furthermore, we propose reducing the production of secondary metabolites by over-expressing galactokinase (GALK1), a bottleneck point in the galactose metabolism.

## Methodology

Two sets of experiments were carried out. In the first set, t-PA producing CHO TF 70R cells were grown in protein and serum free media without glutamine and supplemented with glucose, galactose and glutamate to define two different culture conditions. A high glucose control experiment was performed with 20 mM glucose (G20), and a combined carbon condition with a final concentration of 20 mM of hexose with 6 mM glucose and 14 mM galactose (GG6/14).

Based on these results, transfection of genes involved in galactose metabolism are performed to enhance cell growth by improving galactose metabolism. Cells were transfected with the Galactokinase (GALK1) gene from *Mus musculus*, using Lipofectamine. Experiments were performed to analyze cell proliferation, carbon source consumption lactate production and metabolic flux distribution for the obtained pooled clones, as a preliminary tool to determine if the over-expression of this protein has a positive effect.

In the second culture set, transfected cells were grown in protein free media with 2% Fetal Bovine Serum, supplemented with glucose and galactose to define two different culture conditions with a final concentration of 20 mM of hexose: 6 mM glucose and 14 mM galactose (GalKGG6/14); and 20 mM galactose (Gal20). Extracellular metabolites were measured.

## Results

In combined carbon source experiments, two phases can be distinguished. During the glucose consumption phase cells produce biomass, tPA, lactate and alanine. When glucose is depleted GG6/14 culture enters a second metabolic stage where no significant growth is observed and galactose is consumed along with extracellular lactate and alanine. This is consistent with lactate and alanine being used as a supplementary pyruvate source to support energy metabolism associated with cellular maintenance.

**Figure 1:**
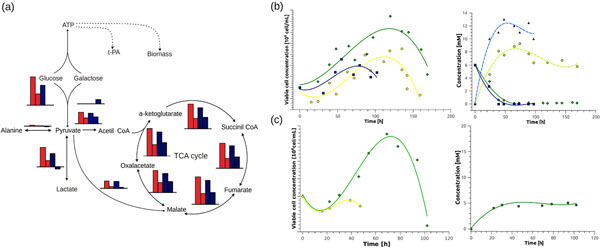
Experimental results and metabolic flux distribution. (a) Metabolic Flux distribution between G20 (red) and GG6/14 (blue) at 50 h and 100 h in culture. Cell density, glucose and lactate concentration in GG6/14  (b) and Gal20 media (c). (▪) CHO tPA,(●) CHO tPA-pcDNA3.1(+), (♦) CHO tPA-GALK1, glucose (▪,♦), lactate (▲,●).

Metabolic flux analysis (MFA) was performed, considering the main reactions of the central glucose and galactose metabolism. Figure 1(a) shows results for two time-points for each culture, an early (50 h) and a late time-point (100 h). For GG6/14, the columns represent glucose and galactose consumption stages, respectively. In the pyruvate node carbon molecules are channeled either towards the TCA cycle, or in the direction of secondary metabolite synthesis such as lactate and alanine. Fluxes from the central carbon metabolism are reduced several times in the late time point for both cultures, except for the Pyr-AcCoA reaction, which plays a central role in the regulation of mammalian metabolism by connecting glycolysis with the TCA cycle. In late culture stages where hexose uptake is low, cell metabolism is directed towards maintaining TCA cycle fluxes in order to obtain energy. To achieve this in GG6/14, galactose and lactate are used as an additional carbon source.

Since galactose consumption appears to be limiting cell growth in GG6/14, there is an open possibility to improve cell growth during galactose consumption while maintaining low lactate production by over-expressing genes involved in galactose metabolism. Transport of galactose has been proposed to explain the low uptake of galactose and the GalK reaction is identified as the limiting step of the galactose metabolism. To achieve this cells are transfected with stable expression vectors to insert the galactokinase GalK1 gene and the galactose transporter Slc2a8.

Obtained clones exhibit a higher growth rate in galactose than control cells due to their ability of metabolize galactose at a increased rate. Transfected cells in GalKGG6/14 consume all available glucose before starting to consume galactose. They also exhibit lower lactate accumulation, indicating that the introduction of these gene does not increase galactose uptake in a manner that would generate secondary metabolites. Control cells are unable to grow on media with galactose as the only carbon source. Transfected cells were able to maintain high viability during 100 hours in this media.

The over-expression of the Slc2a8 (GLUT8) Galactose transporter could enhance the positive effects of the introduction of the galactokinase gene by increasing the availability of substrate at an intracelullar level. Before transfection, mutation of a dileucine motif to alanine is required to allow the expression of this transporter in the plasmatic membrane. For that purpose an strategy using PCR with primers that include this substitutions is currently undergoing.

## Conclusions

When CHO cells are cultured with a combination of glucose and galactose it is possible to achieve extended viability along with a metabolic shift towards lactate consumption, which is triggered when the second hexose is consumed.

Metabolism can be modified through cell engineering to increase cell growth while maintaining low lactate production rates, enabling cells to utilize alternative carbon sources. Specifically this can be achieved with galactose.

## References

[B1] Wlaschin K Hu WS Engineering cell metabolism for high density cell culture via manipulation of sugar transport J of Biotechnol 2007 13 1023 1027 10.1016/j.jbiotec.2007.06.006 17662499

[B2] Altamirano C Illanes A Becerra S Cairó J Gòdia F Considerations on the lactate consumption by CHO cells in the presence of galactose J of Biotechnol 2006 125 547 556 10.1016/j.jbiotec.2006.03.023 16822573

[B3] Neerman J Wagner R Comparative analysis of glucose and glutamine metabolism in transformed mammalian cell lines, insect and primary liver cells J Cell Physiol 1996 166 152 169 10.1002/(SICI)1097-4652(199601)166:1<152::AID-JCP18>3.0.CO;2-H 8557765

